# The plyometric treatment effects on change of direction speed and reactive agility in young tennis players: a randomized controlled trial

**DOI:** 10.3389/fphys.2023.1226831

**Published:** 2023-08-08

**Authors:** Filip Sinkovic, Dario Novak, Nikola Foretic, Jinseo Kim, S. V. Subramanian

**Affiliations:** ^1^ Faculty of Kinesiology, University of Zagreb, Zagreb, Croatia; ^2^ Faculty of Kinesiology, University of Split, Split, Croatia; ^3^ Interdisciplinary Program in Precision Public Health, Department of Public Health Sciences, Graduate School of Korea University, Seoul, Republic of Korea; ^4^ Department of Social and Behavioral Sciences, Harvard T. H. Chan School of Public Health, Boston, MA, United States; ^5^ Harvard Center for Population and Development Studies, Cambridge, MA, United States

**Keywords:** tennis, specific agility, experimental protocol, neuromuscular training, motor abilities

## Abstract

**Aim:** The aim of this paper is to determine the effect of 6 weeks of plyometric training on speed, explosive power, pre-planned agility, and reactive agility in young tennis players.

**Methods:** The participants in this study included 35 male tennis players (age 12.14 ± 1.3 years, height 157.35 ± 9.53 cm and body mass 45.84 ± 8.43 kg at the beginning of the experiment). The biological age was calculated and determined for all participants. 18 of the participants were randomly assigned to the control group, and 17 were assigned to the experimental group. Running speed (sprints at 5, 10, and 20 m), change of direction speed (4 × 10, 20 yards, *t*-test, TENCODS), reactive agility (TENRAG), and explosive power (long jump, single leg triple jump, countermovement jump, squat jump, and single leg countermovement jump) were all tested. The Mixed model (2 × 2) ANOVA was used to determine the interactions and influence of a training program on test results. Furthermore, Bonferroni *post hoc* test was performed on variables with significant time*group interactions.

**Results:** The results of this research indicate that an experimental training program affected results in a set time period, i.e. 5 out of total 15 variables showed significant improvement after experimental protocol when final testing was conducted. The experimental group showed significantly improved results in the 5 m sprint test in the final testing phase compared to the initial testing phase, this was also the case in comparison to the control group in both measurements. Furthermore, the experimental group showed significant improvement in the single leg countermovement jump in the final test, as well as in comparison to the control group in both measurements. The change of direction speed and reactive agility test also exhibited significant improvement in the final testing phase of the experimental group.

**Conclusion:** The results of this research indicated that a 6-week program dominated by plyometric training can have a significant effect on the improvement of specific motor abilities within younger competitive categories. These results offer valuable insights for coaches in designing diverse tennis-specific scenarios to enhance overall performance, particularly focusing on the neuromuscular fitness of their players.

## 1 Introduction

Tennis, as a complex activity, is characterized by a number of specific movement structures that alternate depending on the situation and predominantly require maximum speed over a given period of time (Milanović et al., 2005). Due to the reactive requirements of the game, the total duration of a match, the basis on which it is played and the energy consumption required during a match, it can be said that one of the main goals of training tennis players must be directed towards the development and maintenance of speed, agility and explosive power (Milanović et al., 2005). Pre-planned change of direction speed (CODS) is characterized by a change in the direction of movement that is already known in advance, it is planned and players do not need to react to a certain stimulus. On the other hand, reactive agility (RAG) includes cognitive processing, observational skills, and decision-making factors (Sekulić et al., 2017). In the area of RAG, players most often need to react to a visual stimulus, which is crucial in the field of sports since athletes usually perform agile movements based on visual observation of either the opponent’s motion or the trajectory of the ball (Sekulić et al., 2020). Given that movement in tennis is highly specific, CODS and RAG are considered to be crucial motor abilities (Sekulić et al., 2017; Sekulić et al., 2020).

Regardless of the importance of CODS and RAG in tennis, there are only a few scientific studies dealing with these motor abilities, especially under specific conditions. So far, these abilities have mostly been measured by standardized basic tests. This study will use a reliable and valid tennis-specific change of direction speed test (TENCODS) and a tennis specific reactive agility test (TENRAG) under specific conditions (Sinkovic et al., 2022).

We are increasingly faced with the fact that conditioning training is effective even in the prepubescent phase (Čanaki and Birkić, 2009). Prepuberty should be seen as a time of early anatomical adjustment of the heart, lungs, joints, and muscles to prolonged physical activity (Čanaki and Birkić, 2009). This should serve as the foundation upon which athletes will build aerobic and anaerobic fitness during the specialization and peak performance phase (Čanaki and Birkić, 2009). Although prepubescent conditioning training must be approached with caution, it is clear that dedicating more time to the development ability of changing direction and agility during prepuberty and early puberty increases the chances of fully exploiting this ability’s potential in later stages of sports development (Čanaki and Birkić, 2009). Additionally, it is important to adapt the plan and program of conditioning training during prepubescence and early puberty phases, specifically for the younger competitive categories of tennis players (U-12 and U-14).

Plyometric training offers the necessary stimuli for developing the stretch-shortening cycle (SSC) mechanism and has the potential to improve explosive contractions in both prepubertal and pubertal individuals (Fernandez-Fernandez et al., 2016). In other words, plyometric training focuses on combining strength with speed of movement to generate power (Fernandez-Fernandez et al., 2016). Nowadays, there is increasing exploration of the influence of plyometric training on motor abilities, as well as biomechanical and physiological parameters in tennis (Salonikidis and Zafeiridis, 2008; Kilit and Arslan, 2019). Literature reviews show that plyometric training has the potential to enhance maximal serve velocity and various physical performance components, such as sprint speed, lower extremity muscular power and agility among healthy tennis players (Davies et al., 2015; Novak et al., 2023). Nevertheless, further research is warranted to gather more high-quality evidence regarding the effects of plyometric training on the skill and physical performance of tennis players (Davies et al., 2015). Some studies suggest that regular use of plyometrics in tennis training for younger players has a significant impact on CODS tests (Antekolović et al., 2003; Vuong et al., 2022). The main challenge lies in the lack of appropriate testing mechanisms, as most of the CODS in tennis has been assessed using standardized basic tests or modifications of existing ones, such as the “*t*-test,” “505 test,” and “Spider drill test” (Sinkovic et al., 2022). Additionally, one of the main problems is the lack of research investigating the effect of plyometric training on CODS and RAG in young tennis players, this being something that this study aims to provide answers to. It has been found that CODS is the most influential factor for tennis performance and is strongly influenced by linear speed and jumping power (Vuong et al., 2022). Therefore, it can be concluded that the tests were primarily designed to measure pre-planned agility, where changes in movement direction are planned in advance. It is crucial to emphasize that this study will utilize a specific test to assess reactive agility, which is a key factor for success in tennis.

In accordance with the above, the aim of this paper is to determine the effect of two plyometric, explosive power, pre-planned agility, and reactive agility sessions per week for tennis players from the younger competition categories (U-12 and U-14) in prepuberty and early puberty.

## 2 Methods

### 2.1 Participants

The sample included 35 young male tennis players (age 12.14 ± 1.3 years, height 157.35 ± 9.53 cm and body mass 45.84 ± 8.43 kg at the beginning of the experiment) who were ranked in the top 50 in the National Tennis Association rankings, as well the top 300 on the international “Tennis Europe” rankings. The G-Power program (version 3.1.9.2; Heinrich Heine University, Dusseldorf, Germany) was used to estimate the appropriate number of participants, with an expected effect power of f = 0.33, an alpha level of 0.05, and a statistical power of 0.90. 18 participants were randomly assigned to the control group (CG), and 17 participants were assigned to the experimental group (EG). The biological age was calculated and determined for all participants. To participate in the study, all participants had to meet certain inclusion criteria, including being physically active players who trained for at least 6 hours a week and competed in regional, national, or international tournaments. According to the level of trainability, all participants were at least intermediate or advanced athletes. Exclusion criteria included any injury that would affect tennis play and physical performance at the start of the study. The study was conducted in accordance with the Helsinki Declaration and approved by the Ethics Committee of the Faculty of Kinesiology, University of Zagreb (protocol code 34; date of approval: 13 December 2021). All participants were informed of the research’s purpose and the conditions for participation, and both they and their parents provided prior written consent to participate. The complete testing protocol was explained to them in detail, with a special emphasis on the additional effort required for the research and the risk of injury, which was the same level as during standard training or competition.

### 2.2 Measurements and procedure

The biological age of the participants was assessed through body height (cm), sitting height (cm), body mass (kg), leg length (cm), and chronological age (years). The data obtained was then entered into a specific regression equation for boys to determine PHV maturity offset: −9.236 + (0.0002708 x leg length x sitting height) + (−0.001663 x chronological age x leg length) + (0.007216 x chronological age x sitting height) + (0.02292 x ratio of body mass to body height) (Sinkovic et al., 2023). Therefore, a maturity offset of −1.0 indicates that the player was measured 1 year before peak height velocity, a maturity offset of 0 indicates that the player was measured at the time of peak height velocity, and a maturity offset of +1.0 indicates that the athlete was measured 1 year after peak height velocity. In accordance with this, age at peak high velocity (APHV) was calculated from an estimation between peak height velocity maturity offset and chronological age. The chronological age of the participants (years) was calculated by subtracting the date of birth from the date of measurement. Standing body height (cm) and sitting height (cm) were measured using a portable altimeter (Seca 213; seca gmbh, Hamburg, Germany). Leg length (cm) was calculated by subtracting the sitting height (cm) from the standing height (cm). Body mass (kg) was measured using a portable digital scale (Seca V/700; seca gmbh, Hamburg, Germany), while body fat percentage (%) was measured using the MALTRON BF 900 analyser (Maltron International Ltd., Rayleigh, United Kingdom).

The participants underwent a series of tests to evaluate their speed, agility, and explosive power. Speed assessments included 5, 10, and 20-m sprints, while agility was measured using tests such as the 20-yard run, 4 × 10-yard run, *t*-test, TENCODS, and TENRAG. Explosive power was evaluated through exercises such as the countermovement jump (CMJ), single-leg countermovement jump (CMJ_L, R), squat jump (SJ), long jump (L_JUMP), and single-leg triple jump (SLTJ_L, R). The Powertimer system (Newtest Oy, Oulu, Finland) was used to measure speed, the SportReact system (SportReact, Zagreb, Croatia) for agility, and the Optojump system (Microgate, Bolzano, Italy) for assessing explosive power during jumps. Each test was conducted three times, and the average of the three trials was then calculated for further analysis.

Before the testing session, all participants completed a standardized warm-up specific to tennis. The warm-up consisted of various activities, including light-intensity running covering a distance of 10 × 20 m. Following the running component, participants engaged in dynamic stretching exercises for a total duration of 15 min. These dynamic stretches involved lateral movements, skipping, jumping, lunges, and concluding with four repetitions of sub-maximum acceleration. Subsequently, the participants underwent tests to assess their speed (5, 10, and 20-m sprints), agility (20 yards, 4 × 10 yards, *t*-test, TENCODS, and TENRAG), and explosive power (countermovement jump, one-leg countermovement jump, squat jump, long jump, and one-leg triple jump).

#### 2.2.1 Linear sprint speed tests

For the linear sprint speed tests, three electronic timing gates were positioned at predetermined distances of 5, 10, and 20 m from a designated starting line. Participants were instructed to their preferred foot positioning, placed on a marked line on the floor, and initiate the sprint from a stationary standing start. Their objective was to cover the 20-m distance as quickly as possible. Timing measurements were recorded at the 5-m mark (using the first electronic timing gate), the 10-m mark (using the second electronic timing gate), and the 20-m mark (using the third electronic timing gate). Each participant performed three trials, with a 3–4-min rest period between each trial. The mean value of the three trials was calculated for further analysis (Sinkovic et al., 2023).

#### 2.2.2 Explosive power tests

During the countermovement and single-leg countermovement jump tests, participants kept their hands positioned on their hips to minimize any impact from the upper body on jump performance. Starting from a standing position with knees straight, participants performed a squat motion, lowering themselves to approximately a 90°, and then rapidly accelerated in a vertical direction using both legs or a single leg. Each participant completed three trials of the tests, with a 1-min rest period between each trial. The mean value of the three trials was then calculated for further analysis (Sinkovic et al., 2023).

In the squat jump test, participants started with a knee flexion angle of 90°, maintaining a straight torso, hands on hips, and feet positioned shoulder-width apart. They held this position for 2 s before initiating the jump. The push-off phase of the jump was performed without any form of countermovement. During the highest point of the jump, participants fully extended their legs. The landing phase involved both feet landing together in an upright position, with knees fully extended. Each participant completed three trials of the test, with a 1-min rest period between each trial. The mean value of the three trials was then calculated for further analysis (Sinkovic et al., 2023).

In the long jump test, participants were provided with standardized instructions to perform a long jump starting from a standing position. They were allowed to initiate the jump with bent knees and utilize arm swinging for assistance. A marked line on a hard surface served as the starting point, and the length of the jump was measured using a tape affixed to the floor. Each participant completed three trials of the test, with a 1-min rest period between each trial. The mean value of the three trials was then calculated for further analysis (Sinkovic et al., 2023).

In the single-leg triple jump test, participants began by standing on one designated leg, with their toe positioned on the starting line. When ready, they performed three consecutive maximal jumps forward using the designated leg. Upper extremity movement during the single-leg horizontal hop was not restricted, although participants were instructed to land firmly on the last jump. After practice trials, three test trials were conducted on each leg in alternating order. A 30-s rest period was allowed between practice and test trials. The mean distance of the three test trials for each leg was then calculated for further analysis (Sinkovic et al., 2023).

#### 2.2.3 Change of direction speed and reactive agility tests

In the 20-yard test, participants assumed a three-point stance and sprinted 5 yards in one direction, followed by a 10-yard sprint in the opposite direction, and then returned to the starting point. This test evaluates lateral speed and coordination. The timing commenced upon a sound of the signal and concluded when the participant crossed the timing gate upon their return. The time was measured in hundredths of a second (Sinkovic et al., 2023).

In the 4 × 10 yard test, parallel lines were marked on the floor with a distance of 10 yards between them. Participants were required to shuttle back and forth four times between the starting line and the other line as fast as possible, ensuring that both feet crossed each line during each run. The timing started upon a sound of the signal and ceased when the participant crossed the timing gate upon their return. The time was measured in hundredths of a second (Sinkovic et al., 2023).

In the *t*-test, a configuration of four cones was arranged in the shape of a “T.” The starting cone was placed 9.14 m away from the first cone, and two additional cones were positioned 4.57 m from either side of the second cone. An electronic timing gate measuring 0.75 m in height and 3 m in width was set up in alignment with the marked starting point. Participants were instructed to sprint forward from the start line to the first cone (9.14 m) and touch it with their right hand. They then shuffled 4.57 m to the left to the second cone, touching it with their left hand. Next, they shuffled 9.14 m to the right to the third cone, touching it with their right hand, and then shuffled 4.57 m back to the middle cone, touching it with their left hand. Finally, they backpedaled to the start line. The timing commenced upon a sound of the signal and concluded when the participant crossed the timing gate upon their return. Trials were considered unsuccessful if participants did not touch a designated cone, crossed their legs during shuffling, or failed to face forward throughout the test. The time was measured in hundredths of a second (Sinkovic et al., 2023).

**FIGURE 1:**
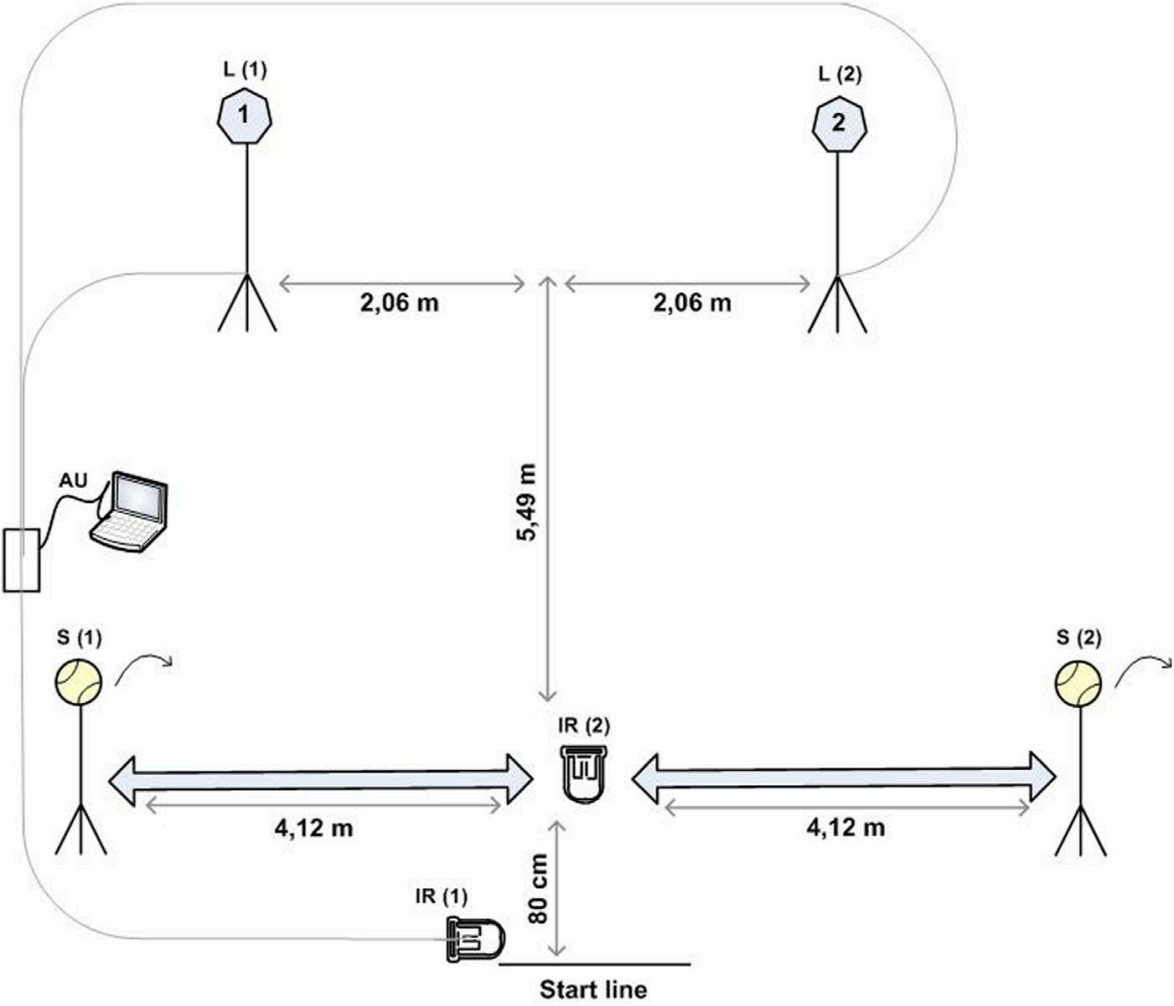
TENCODS and TENRAG test.

The change of direction speed (TENCODS) and reactive agility (TENRAG) variables were assessed using tests that exhibit excellent metric properties and are both reliable and valid (Sinkovic et al., 2022). The reliability of the pre-planned agility tests is slightly higher (CA = 0.92 and 0.92; ICC = 0.86 and 0.82) compared to the reactive agility tests (CA = 0.90 and 0.89; ICC = 0.74 and 0.72) (Sinkovic et al., 2022). The SportReact system (SportReact, Zagreb, Croatia) was used to measure these tests, which consists of laser tape sensors and LED screens displaying differing signs and colors (Sinkovic et al., 2022). The TENCODS and TENRAG tests are designed to simulate specific movements in tennis (Figure 1). Participants start from a predetermined starting line, and the timing begins when the infrared signal (IR1) next to the starting line is interrupted by the “split step.” At this point, one of the two lights (L1 or L2) illuminates, the participant must identify which light is lit, and perform a run with overstepping and a lateral side-to-side technique to reach a stand with a ball (S1 or S2) and hit the ball with a forehand or backhand stroke in front of their body with sufficient force for the ball to hit the ground. After playing the shot, the player should quickly return to the device in front of the starting line, interrupting the infrared signal (IR2), which stops the measurement. In the TENCODS test, participants are aware in advance of which light will illuminate, allowing them to plan their running and shot execution. Each test was performed nine times, with a 60-s break between measurement repetitions, and the mean value of the measurements was then used for further analysis (Sinkovic et al., 2022).

### 2.3 Study design

After the initial testing of the participants motor abilities, the control group (CG), in addition to standard technical-tactical training, continued with regular conditioning training that included a combination of strength exercises, plyometrics and agility drills. There were four standard technical-tactical training sessions and two conditioning training sessions per week. All participants were familiarized with the tests prior to the main test session. The exercises are arranged in a way that allows for a proper warm-up, progression, and targeted muscle group activation (Table 1). The experimental group (EG) underwent a 6-week plyometric training program in addition to their regular technical-tactical training sessions (Table 2). This combination ensured that both groups had an equal total load volume, the same number of training sessions, and an equal duration of training. To ensure that the participants followed the same training program, despite belonging to different clubs, licensed tennis coaches were involved in the study. The study design incorporated the assistance and supervision of these coaches to ensure consistency in training implementation. To guarantee that both groups followed the same volume of training, the licensed tennis coaches closely monitored the training sessions of each group. They provided instructions and guidance to the participants, ensuring that the prescribed training volume was adhered to by both groups. The coaches maintained regular communication with the researchers to report any deviations or inconsistencies in training volume. By involving licensed tennis coaches, who were experienced and knowledgeable in training methodologies, the study aimed to minimize variations in training implementation and ensure that both groups received similar training volumes throughout the study period. Based on the expert literature, previous research and recommendations from licensed tennis coaches, the plyometric training exercises were selected for this program. The execution of the plyometric training exercises was carefully described and explained to the participants before the training sessions. Licensed tennis coaches provided detailed instructions on the technical execution of each exercise. They also used demonstrations and visual examples to show participants the correct technique. Participants were given the opportunity to practice each exercise under the supervision of the coaches to ensure proper execution of the movements and understanding of the technique. Coaches provided feedback and corrected any errors or deficiencies in exercise execution to ensure safe and effective implementation of the plyometric training. The training schedule, as shown in Table 1 and Table 2, outlined the number of weeks, exercise names, sets and repetitions, and rest periods. The participants typically performed 3 to 4 sets of 4–6 exercises, with each exercise consisting of 5–10 repetitions. They were instructed to exert maximum effort during all exercises. The rest periods between sets ranged from 15 to 60 s, while the rest periods between exercises varied from 60 to 120 s. The training sessions lasted between 30 and 45 min, including the warm-up period, and were supervised by a certified strength and conditioning coach (Novak et al., 2023). The plyometric program includes unilateral and bilateral jumps, both vertical and horizontal. The training plan was programmed based on the principle of a progressive increase in load volume, which was measured by the weekly increase in the number of jumps and the ratio between the number of bilateral and unilateral jumps per week. Each training session consisted of a preparatory, main, and final part, and there was a minimum of 48 h between two training sessions. At the end of the experiment, a final test was conducted to determine the effects of plyometric training on motor abilities, pre-planned change of direction speed, and reactive agility.

**TABLE 1:** Basic conditioning training program.

Training week	Exercise	Sets x reps	Pause (s)
1	Plank	3 × 30 s	30–60/90–120
	Leg raises	3 × 10	30–60/90–120
	Squat jumps	3 × 10	30–60/90–120
	Push-ups	3 × 10	30–60/90–120
2	Countermovement jump	3 × 6	30–60/90–120
	Hurdle hops forward (20–30 cm)	3 × 6	30–60/90–120
	Sprint 20 m	4 × 1	30–60/90–120
	Sprint 40 m	4 × 1	30–60/90–120
3	Plank	3 × 30 s	15–30/90
	Lunges: 3 sets x 10 reps (each leg)	3 × 10 (each leg)	15–30/90
	High knees	3 × 20 s	15–30/90
	Russian twists	3 × 10 (each side)	15–30/90
4	Squats	3 × 8	30–60/90–120
	Assisted pull ups	3 × 6	30–60/90–120
	Dumbbell bicep curls	3 × 10	30–60/90–120
	Bicycle crunches	3 × 15 (each side)	30–60/90–120
5	Agility ladder drills	3 × 10	30–60/90–120
	Shuttle runs	3 × 4	30–60/90–120
	Vertical jumps	3 × 4	30–60/90–120
	Medicine ball slams	3 × 8	30–60/90–120
6	Plank	3 × 30 s	15–30/90
	Mountain climbers	3 × 10	15–30/90
	Box jumps	3 × 4	15–30/90
	Tricep dips	3 × 8	15–30/90

**TABLE 2:** Six-week plyometric training program.

Training week	Exercise	Sets x reps	Pause (s)
1	Ankle cone hops	3 × 10	15–30/90
	Ankle cone hops side to side	3 × 10	15–30/90
	Countermovement jumps	4 × 5	15–30/90
	Broad jumps	4 × 5	15–30/90
2	1-leg ankle hops forward	3 × 10	30–60/90–120
	Countermovement jumps	3 × 8	30–60/90–120
	Continious broad jumps	3 × 2 × 3	30–60/90–120
	Lateral bounds + stick	3 × 6	30–60/90–120
	2–1 Hurdle hops forward (20–30 cm)	3 × 10	30–60/90–120
3	1-leg ankle hops lateral	3 × 10	30–60/90–120
	Countermovement jump	3 × 10	30–60/90–120
	1:2 broad jumps	3 × 4	30–60/90–120
	Zig zag bounds + stick	3 × 8	30–60/90–120
	2–1 Hurdle hops lateral (20–30 cm)	3 × 10	30–60/90–120
4	1-leg square ankle hops	3 × 8	30–60/90–120
	1-leg Countermovement jump	3 × 5	30–60/90–120
	Continious broad jumps	3 × 3 × 3	30–60/90–120
	Lateral bounds (1-1-stick)	3 × 8	30–60/90–120
	2–1 Multidirectional hurdle hops	3 × 10	30–60/90–120
5	1-leg square ankle hops	3 × 12	30–60/90–120
	1-leg Countermovement jump	3 × 5	30–60/90–120
	1:2 Broad jumps	3 × 8	30–60/90–120
	Zig zag bounds (1-1-stick)	3 × 10	30–60/90–120
	2–1 Multidirectional hurdle hop	3 × 10	30–60/90–120
6	Ankle cone hops	3 × 10	15–30/90
	Ankle cone hops side to side	3 × 10	15–30/90
	Countermovement jump Broad jumps	4 × 5	15–30/90
	Broad jumps	4 × 5	15–30/90

### 2.4 Statistical analysis

All statistical analysis was performed using R Statistical Software (version 4.2.2 R Foundation for Statistical Computing, Vienna, Austria). The normality of the distribution was tested with the Shapiro-Wilk W test. Descriptive statistics were used to determine the basic parameters of test results for each group (CG and EG) in initial and final testing phases (mean - $\bar{x}$ ; standard deviation—SD). Mixed model (2 × 2) ANOVA was used to determine interactions and the effect of the training program on test results. Maturity status calculated based on PHV maturity offset was included as a covariate. The partial ŋ2 coefficient was used as an indicator of effect size. Furthermore, when the group effect was significant, the paired *t*-test with Bonferroni correction was used for a *post hoc* analysis. For the sensitivity analysis, a complex variance model was conducted to evaluate the heterogeneity of the intervention on 15 different outcomes. It means that the constant variance assumption was loosened to allow for differential variance in outcomes to be estimated by the intervention status. This modelling offers information about the magnitude and direction of the effect on variance. Complex variance models were fitted by partitioning the level-1 variance according to the intervention status (intervention group variance ( $\sigma_{e1}^{2}$ ), and control group variance ( $\sigma_{e2}^{2}$ )):

$$Y_{i}=\beta_{0}+\beta_{1}{Intervention}_{i}+e_{1i}{Intervention}_{i}+e_{2i}{Control}_{i}$$

For this model, the residual distribution of the specified model:

$$\left[\begin{array}{c} e_{1i}\\ e_{2i} \end{array}\right]∼N\left(0,\left[\begin{array}{cc} \sigma_{e1}^{2} & \\ - & \sigma_{e2}^{2} \end{array}\right]\right)$$

Where $Y_{i}$ represents the 15 different outcomes, ${Intervention}_{i}$ is an indicator variable for the intervention group ( ${Intervention}_{i}=1$ if one was in the intervention group) and ${Control}_{i}$ is an indicator variable for the control group. Due to the limited number of observations, the point estimation was calculated through likelihood procedures (IGLS) and then applied parametric, bias-corrected bootstraps for the fitted models, with replicate set size set to 100, max iterations per replicate set to 25, and the maximum number of sets set to 5. This procedure ran by MLwiN Version 3.05. (Centre for Multilevel Modelling, University of Bristol). The level of statistical significance was set at *p* < 0.05 and the confidence interval was 95%.

## 3 Results

Basic descriptive parameters of the test results were calculated and are presented in Table 3. Additionally, time*group interactions were determined for all variables. Significant interactions were observed in the sprint test results for split times at 5 m (F = 7.80; *p* = 0.00) and 10 m (F = 5.76; *p* = 0.02), indicating differences between the groups over time. However, there were no significant interactions found in the 20 m sprint test results. The best average acceleration results at 5 and 10 m were obtained in the final testing of the, EG, while the best average results at 20 m were found in the final testing of the CG. The 4 × 10 yards and 20 yards CODS tests did not show significant interactions, with the best average results achieved in the final testing of the, EG.

**TABLE 3:** Results (Mean ± SD) of Intervention Group and Control Group Before and After the intervention Using 2 × 2 Mixed Analysis of Variance (ANOVA).

Variable	Control group		Experimental group		Interaction Time*Group			Post hoc bonferroni test	
	Initial testing	Final testing	Initial testing	Final testing					
	$\bar{x}$ ±SD	$\bar{x}$ ±SD	$\bar{x}$ ±SD	$\bar{x}$ ±SD	F	p	Partial η2	Comparison	p
Sprint 5 m (s)	1.27 ± 0.05	1.27 ± 0.09	1.27 ± 0.07	1.19 ± 0.06	7.8	0.01	0.2	CG > EG	0.01
Sprint 10 m (s)	2.12 ± 0.09	2.12 ± 0.12	2.14 ± 0.1	2.07 ± 0.1	5.76	0.02	0.15	CG > EG	0.23
Sprint 20 m (s)	3.71 ± 0.15	3.63 ± 0.24	3.79 ± 0.21	3.68 ± 0.21	0.12	0.74	0.00	-	-
CODS 4 × 10 yards (s)	10.62 ± 0.63	10.46 ± 0.49	10.64 ± 0.55	10.29 ± 0.45	1.57	0.22	0.05	-	-
CODS 20 yards (s)	5.65 ± 0.29	5.6 ± 0.28	5.62 ± 0.35	5.46 ± 0.25	2.26	0.14	0.07	-	-
CODS *t*-test (s)	12.22 ± 0.64	11.87 ± 0.63	12.14 ± 0.8	11.56 ± 0.69	3.28	0.08	0.09	-	-
Long jump (cm)	162.85 ± 16.48	165.2 ± 16.95	155.55 ± 18.18	172.8 ± 20.23	33.03	0.00	0.51	CG < EG	0.236
Triple jump_L (cm)	459.76 ± 57.44	462.93 ± 51.7	427.35 ± 65.32	461.04 ± 66.14	15.1	0.00	0.32	CG < EG	0.925
Triple jump_R (cm)	454.11 ± 56.26	451.24 ± 60.06	442.67 ± 66.39	471.14 ± 65.24	28.34	0.00	0.47	CG < EG	0.35
CMJ (cm)	23.3 ± 3.37	23.58 ± 3.34	21.56 ± 3.31	24.41 ± 3.57	28.96	0.00	0.48	CG < EG	0.48
SJ (cm)	23.1 ± 3.81	23.31 ± 3.91	21.48 ± 3.87	23.61 ± 3.86	15.9	0.00	0.33	CG < EG	0.82
CMJ_L (cm)	11.52 ± 1.57	11.73 ± 1.52	10.79 ± 1.13	12.97 ± 1.82	17.37	0.00	0.35	CG < EG	0.04
CMJ_R (cm)	11.54 ± 1.11	11.37 ± 1.37	11.21 ± 1.71	13.01 ± 2.01	22.39	0.00	0.41	CG < EG	0.01
TENCODS (s)	3.2 ± 0.17	3.18 ± 0.15	3.23 ± 0.16	3.04 ± 0.11	26.5	0.00	0.45	CG > EG	0.01
TENRAG (s)	3.38 ± 0.19	3.34 ± 0.19	3.36 ± 0.13	3.16 ± 0.17	19.08	0.00	0.37	CG > EG	0.01

Legend: CMJ (cm)—countermovement jump with arms set on hips; SJ (cm)—squat jump; CMJ_L (cm)—single leg (left) countermovement jump with arms set on hips; CMJ_R (cm)—single leg (right) countermovement jump with arms set on hips; TENCODS (s)—change of direction speed test; TENRAG (s)—reactive agility test; *—significant interaction (*p* < 0.05).

In tests assessing horizontal jump performance (standing long jump, triple jump_L, triple jump_R), the lowest average results were measured in the initial testing, and the best results were achieved in the final testing of the, EG. Significant interactions were also observed for these tests (*p* = 0.00). Regarding vertical jump performance, the triple jump_L and triple jump_R tests showed the lowest values in the initial testing and the best average values in the final testing of jump height in the, EG. Significant interactions were determined for all vertical-oriented jump tests (*p* = 0.00). Single leg countermovement jump (CMJ) tests yielded better results when performed with the right leg (13.01 cm).

Furthermore, the change of direction speed (TENCODS) and reactive agility (TENRAG) results also exhibited significant interactions (TENCODS - F = 26.50; *p* = 0.00 and TENRAG—F = 19.08; *p* = 0.00), with the best results being measured in the final testing of the, EG.

In addition, for all variables that were significant in 2 × 2 (time*group) an ANOVA, Bonferroni *post hoc* test (Table 3) was performed to further determine differences in each variable. The, EG showed significantly improved results in the 5 m test in the final testing compared to the initial testing, as well as in comparison to the control group in both measurements. There were no significant differences in the 10 and 20 m sprint results between the initial and final testing within the, EG. T-tests revealed no significant differences between the initial and final testing for both the CG and the, EG. Significant differences were not observed in the, EG between the initial and final testing in tests focusing on horizontal jump ability (single leg triple jump) and vertical tests performed with both legs (countermovement jump and squat jump). Furthermore, the, EG showed significant improvement (*p* = 0.01) in the single leg countermovement jump in the final testing. The change of direction speed and reactive agility test also exhibited significant improvement in the final testing of the, EG (*p* = 0.01).

**FIGURE 2:**
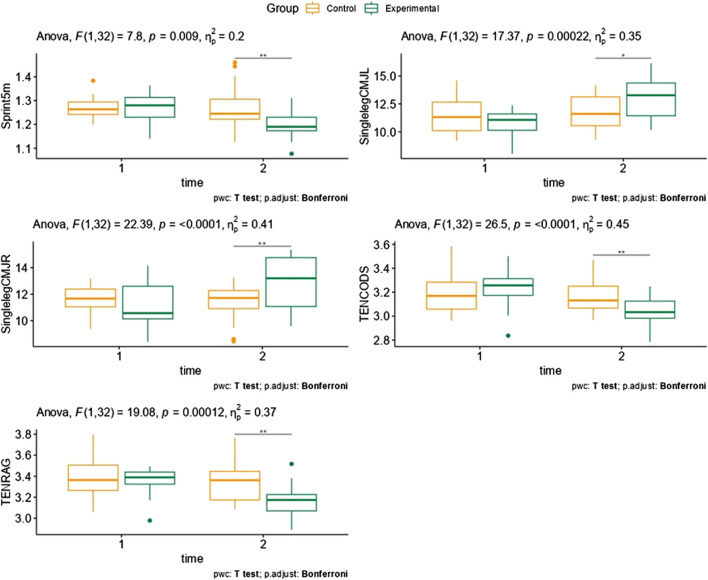
Box plots of selected variables that indicated statistically significant for 2 × 2 Mixed Analysis of Variance (ANOVA) and paired T tests.

The results of this research indicate that an experimental training program affected results in a set time period, i.e. 5 out of total 15 variables showed significant improvement after experimental protocol when final testing was conducted. Moreover, modelling variance by intervention status revealed that plyometric intervention indeed improved the agility, meaning that substantial heterogeneity treatment effect exists and supporting results from 2*2 mixed ANOVA. (Figure 2). displays box plots of selected variables that showed statistically significant differences in the 2 × 2 Mixed Analysis of Variance (ANOVA) and paired t-tests.

Table 4 represents the results of partitioned variance in different outcomes when assuming different variances (heterogeneity) by intervention status (level-1). When we allow complex level-1 heterogeneity by intervention status, we observed everything bar two outcome variables (CMJ and sprint 20 m), the variance of the other outcomes did not differ by the intervention status. On the one hand, the CMJ variable indicated the presence of heterogeneous treatment effect $\left(\sigma_{Intervention\;Group}^{2}\left[\pm 1.96*SE\right]=3.825\left[1.542;6.108\right],\sigma_{Control\;Group}^{2}\left[\pm 1.96*SE\right]=0.789\left[0.266;1.312\right]\right)$ . On the other hand, the sprint 20 m variable showed the treatment reached the control group heterogeneously rather than the intervention group $\left(\sigma_{Intervention\;Group}^{2}\left[\pm 1.96*SE\right]=0.005\left[0.001;0.009\right],\sigma_{Control\;Group}^{2}\left[\pm 1.96*SE\right]=0.045\left[0.014;0.076\right]\right)$ . Lastly, since the ANOVA requires the homogeneity of variances, the sensitivity analysis suggests that the main analysis sufficed one of the primary assumptions in ANOVA.

**TABLE 4:** Heterogeneity by intervention status (*n* = 35).

Variable	Intervention group	Control group	Difference in variance
Sprint 5 m (s)	0.003 [0.001; 0.005]	0.009 [0.003; 0.015]	−0.006
Sprint 10 m (s)	0.004 [0.002; 0.006]	0.01 [0.004; 0.016]	−0.006
Sprint 20 m (s)	0.005 [0.001; 0.009]	0.045 [0.014; 0.076]	−0.040
CODS 4 × 10 yards (s)	0.109 [0.031; 0.187]	0.153 [0.049; 0.257]	−0.044
CODS 20 yards (s)	0.028 [0.008; 0.048]	0.047 [0.012; 0.082]	−0.019
CODS *t*-test (s)	0.131 [0.035; 0.227]	0.094 [0.023; 0.165]	0.037
Long jump (cm)	99.085 [34.236; 163.934]	37.726 [13.455; 61.997]	61.359
Triple jump_L (cm)	779.875 [212.82; 1346.93]	242.289 [87.394; 397.184]	537.586
Triple jump_R (cm)	322.594 [122.892; 522.296]	387.5 [164.476; 610.524]	−64.906
CMJ (cm)	3.825 [1.542; 6.108]	0.789 [0.266; 1.312]	3.036
SJ (cm)	3.292 [0.587; 5.997]	1.015 [0.325; 1.705]	2.277
CMJ_L (cm)	2.855 [0.985; 4.725]	0.98 [0.27; 1.69]	1.875
CMJ_R (cm)	2.316 [0.85; 3.782]	1.168 [0.37; 1.966]	1.148
TENCODS (s)	0.009 [0.003; 0.015]	0.007 [0.001; 0.013]	0.002
TENRAG (s)	0.015 [0.005; 0.025]	0.005 [0.001; 0.009]	0.010

Legend: CMJ (cm)—countermovement jump with arms set on hips; SJ (cm)—squat jump; CMJ_L (cm)—single leg (left) countermovement jump with arms set on hips; CMJ_R (cm)—single leg (right) countermovement jump with arms set on hips; TENCODS (s)—change of direction speed test; TENRAG (s)—reactive agility test.

## 4 Discussion

The findings of this study provide strong evidence for the positive effects of a 6-week plyometric training program on motor abilities in young tennis players. The, EG demonstrated significant improvements in various aspects compared to the CG, indicating the effectiveness of the training program. Specifically, the, EG showed enhanced sprint performance at 5 m, indicating improved acceleration and speed. These improvements in sprint times highlight the positive impact of the plyometric training program on the players’ explosive power and running abilities. While no significant interactions were found in the 10 and 20 m sprint test, it is important to note that the, EG still achieved better average results in the final testing phase compared to the CG. The, EG also exhibited notable advancements in jump performance. Significant interactions were observed in horizontal jump tests, with the best results being achieved in the final testing phase. This indicates that the plyometric training enhanced the players’ ability to effectively generate power and explosiveness in horizontal jumps. Additionally, significant interactions were found in vertical jump tests, particularly in the triple jump tests, where the, EG showed improved jump heights in the final stage of testing. The results suggest that the plyometric exercises positively influenced the players’ vertical jump performance, contributing to their overall jumping abilities. Moreover, the, EG displayed significant improvements in change of direction speed and on the reactive agility tests. These findings indicate that the plyometric training program enhanced the players’ ability to change direction quickly and react to unpredictable movements, both of which are crucial in tennis. Sensitivity analysis manifested the main analysis’s assumption. Hence, it was an adequate analysis. Since pre-planned change of direction speed (CODS) and reactive agility (RAG) are distinct and separate abilities influenced by multiple factors, this study represents an innovative research effort in tennis, providing valuable insights into the effects of plyometric training on both aspects. Possible reasons for a slightly greater influence on CODS comes down to the simple fact that there is no decision-making factor in these tests. The movement structure is known in advance, so the participants are less susceptible to the influence of errors during execution. While on the other hand, the RAG test is considered more complex and more difficult to perform due to the greater demand on reaction speed. It is logical that the result will be somewhat weaker. It can be concluded that RAG is influenced not only by motor abilities but also by many other cognitive factors such as observation, perception, anticipation, or decision-making speed. The plyometric training program used in this research should be employed in tennis due to its potential benefits in enhancing specific physical qualities and skills required in the sport, such as improved power and explosiveness, enhanced agility and quickness, increased speed and acceleration, enhanced lower-body strength and injury prevention. Such plyometric training program can lead to several neuromuscular adaptations that contribute to improved athletic performance. These adaptations include enhanced motor unit recruitment and synchronization, improved intermuscular coordination, increased muscle fiber activation and force production, enhanced stretch-shortening cycle utilization, and improved proprioception and reactive capabilities. These neuromuscular adaptations can result in increased power output, greater force absorption and production during explosive movements, improved movement efficiency, and enhanced overall athletic performance (Fatouros et al., 2000; Chimera et al., 2004; Markovic et al., 2007).

Plyometric training is increasingly being researched as a beneficial tool for improving motor abilities in tennis players. Several studies have examined the effects of plyometric training on tennis players and have reported positive changes in their athletic performance. In a study conducted by Sadić et al. (2011), plyometric training was found to have a positive impact on the physical fitness of young tennis players. Improvements were observed in strength, speed, and agility. Granacher et al. (2016) also investigated the effects of plyometric training in prepubescent tennis players. The results showed that plyometric training led to improvements in physical fitness, including strength, speed, and agility.

In another study by Kovacs (2006), it was found that plyometric training can enhance serve performance in pubescent boys. This study highlights the potential of plyometric training in improving specific aspects of tennis gameplay. Plyometric training has also been studied in relation to maximal power output in tennis players. Other research has focused on the effects of plyometric training on acceleration and agility in young tennis players. Čavala et al. (2017) investigated the effects of plyometric training on acceleration and agility and found positive changes in these motor abilities in young tennis players. These studies suggest that plyometric training can have a positive influence on motor abilities in tennis players. Improvements have been observed in strength, speed, agility, and specific aspects of tennis gameplay such as serving. However, it is important to note that individual responses to training may vary, and it is necessary to tailor the training program to individual needs and goals of the tennis players. Similar results have been obtained in research carried out on young soccer players (10–14 years old) where plyometric training for 6 weeks significantly improved agility results (Ramírez-Campillo et al., 2015). Other studies have reported improvements in the Illinois Agility Test scores after 7 weeks of low-volume plyometric training (Ramírez-Campillo et al., 2014). Previous studies have also shown significant changes in the Illinois Agility Test score after 8 and 12 weeks of plyometric training in prepubescent soccer players (Negra et al., 2020). Some research studies have connected the plyometric training program with the ability to change direction, reporting improvements in agility test times after 6 weeks of training (Miller et al., 2006). Similar results have been observed in handball and basketball players with an average age 22.5 ± 0.4 years, where 8 weeks of plyometric training led to decreased agility test times (Bal et al., 2011; Rameshkannan and Chittibabu, 2014). Meta-analysis studies on pubertal and young athletes have reported improvements in agility indicators by 2%–5% after the implementation of plyometric training (Markovic and Mikulic, 2010). While the present study provides evidence for the positive effects of a 6-week plyometric training program on motor abilities in young tennis players, it is important to acknowledge that there are some contradictions with previous studies. Radnor et al. (2020) conducted a systematic review and meta-analysis on neuromuscular training interventions in youth sports, including plyometric training. While they acknowledged some positive effects, they also highlighted limited evidence and inconsistent findings in regards to motor ability improvements. Similarly, Khlifa et al. (2010) investigated the effects of plyometric training with and without added load on jumping ability in basketball players. Their findings suggested that plyometric training without added load did not result in significant improvements in jumping ability compared to the control group, indicating a lack of positive effects. Furthermore, Spurrs et al. (2003) explored the impact of plyometric training on distance running performance. Their study concluded that plyometric training alone may not significantly improve distance running performance, suggesting that the effects of plyometrics may vary depending on the specific motor ability being targeted. These studies present alternative findings and indicate that the effects of plyometric training may not be universally positive for all motor abilities and sports. It highlights the need for further research to understand the specific contexts and factors that influence the effectiveness of plyometric training. Despite these contradictions, it is worth noting that the overall body of research still supports the positive effects of plyometric training on motor abilities in various sports. However, individual responses and specific contexts should be considered when implementing plyometric training programs.

Summarizing the results of previous research, plyometric training aimed at developing the ability to change direction has been conducted with different age categories for durations ranging from 6 to 12 weeks. However, agility is now classified as consisting of two branches: pre-planned change of direction speed, where the change of movement direction is known in advance, and reactive agility, which includes a cognitive component involving observation and decision-making factors (Zeljko et al., 2020). With the advancement of sports science, more studies have focused on determining the reliability of tests for assessing pre-planned change of direction speed and reactive agility. These tests are often specific, aiming to replicate situations within a chosen sport. Such research has been conducted on various athlete samples including Australian football players (Henry et al., 2013), rugby players (Green et al., 2011), netball players (Farrow et al., 2005), basketball players (Sisic et al., 2016; Sekulić et al., 2017), soccer players (Knoop et al., 2013; Gilić et al., 2019; Krolo et al., 2020), and futsal players (Benvenuti et al., 2010; Sekulić et al., 2019; Sekulić et al., 2020; Zeljko et al., 2020).

This study has certain limitations that should be considered. Firstly, the participants in this research were young tennis players in a highly sensitive and crucial stage of development. Additionally, the motor tests were conducted using a convenience sample of subjects under controlled conditions. Therefore, further longitudinal investigations are needed to thoroughly examine the impact of biological age on motor abilities in young tennis players. This research suggests that coaches and practitioners can effectively use a plyometric training program to enhance the desired physical fitness in young tennis players. Specifically, the study demonstrated that plyometric training had a greater impact on pre-planned change of direction agility rather than reactive agility, highlighting the need for including cognitive training in the development of reactive agility. Coaches should take into consideration the variations in physical performance and the practical implications of maturation when planning the long-term development of young tennis players. Future research should aim to include participants of different genders and competition categories, subsequently enabling the acquisition of more precise and comprehensive data for an enhanced practical and scientific contribution. Such information would prove valuable to coaches in designing specific conditioning strategies to foster the motor characteristics of young tennis players.

## 5 Conclusion

The results of this research indicated that a 6-week program dominated by plyometric training can have a significant effect on the improvement of specific motor abilities in the younger competitive categories of tennis players (U-12 and U-14). It is especially important to emphasize how the training programs impacted both abilities, namely, change of direction speed and reactive agility. However, it is important to note that there are risks and dangers associated with plyometric training, particularly for young athletes in the prepubescent and early puberty stages. This primarily pertains to moderately complex plyometric exercises that may lead to acute injuries or various overexertion syndromes. Therefore, it is crucial to adhere to methodological principles and progressively advance from simpler to more complex exercises when implementing plyometric content. Our results offer valuable insights for coaches in designing diverse tennis-specific scenarios to enhance overall performance, particularly focusing on the neuromuscular fitness of their players. Further research is required to explore interventions that can effectively improve sport-specific neuromuscular fitness, with the ultimate aim of enhancing overall performance.

## Data Availability

The raw data supporting the conclusion of this article will be made available by the authors, without undue reservation.
